# S- and N-Co-Doped Carbon-Nanoplate-Encased Ni Nanoparticles Derived from Dual-Ligand-Assembled Ni-MOFs as Efficient Electrocatalysts for the Oxygen Evolution Reaction

**DOI:** 10.3390/molecules30040820

**Published:** 2025-02-10

**Authors:** Huijuan Han, Yalei Zhang, Chunrui Zhou, Haixin Yun, Yiwen Kang, Kexin Du, Jianying Wang, Shujun Chao, Jichao Wang

**Affiliations:** 1 School of Chemistry and Chemical Engineering, Henan Institute of Science and Technology, Xinxiang 453003, China; zhangyaleihist@163.com (Y.Z.); zhouchunruihist@163.com (C.Z.); haixinyun2025@163.com (H.Y.); dukexinhist@163.com (K.D.); 2 Henan Provincial Ecological Environment Monitoring and Safety Center, Department of Ecology and Environment of Henan Province, Zhengzhou 450046, China; 3 Xinxiang Engineering Technology Research Center of Functional Medical Nanomaterials, School of Basic Medical Sciences, Xinxiang Medical University, Xinxiang 453003, China

**Keywords:** metal–organic frameworks, heteroatom-doped electrocatalysts, oxygen evolution reactions

## Abstract

To achieve the “double carbon” goal, it is urgent to reform the energy system. The oxygen evolution reaction (OER) is a vital semi-reaction for many new energy-storage and conversion devices. Metal nanoparticles embedded in heteroatom-doped carbon materials prepared by the pyrolyzing of metal–organic frameworks (MOFs) have been a key route to obtain high-performance electrochemical catalysts. Herein, a nanocatalyst embedding Ni nanoparticles into S- and N-co-doped carbon nanoplate (Ni NPs@SN-CNP) has been synthesized by pyrolysis of a Ni-MOF precursor. The prepared Ni NPs@SN-CNP exhibits superior oxygen evolution performance with an overpotential of 256 mV to attain 10 mA cm^−2^ and a low Tafel slope value of 95 mV dec^−1^. Moreover, a self-assembled overall-water-splitting cell with Ni NPs@SN-CNP/NF||Pt-C/NF achieves a low potential of 1.56 V at 10 mA cm^−2^ and a high cycling stability for at least 10 h. The improvement in this performance is benefit from its large surface area, unique morphology, and the nanostructure of the electrocatalyst. This study presents a novel and simple approach to designing high-performance OER catalysts.

## 1. Introduction

Because of the environmental pollution and energy shortage, all the countries in the world began to study solar energy, hydrogen energy, wind energy, and ocean energy instead of the traditional fossil fuels [1,2,3]. During these new energies, hydrogen energy is the most promising to solve the problems of environmental pollution and resource shortage of secondary energy, therefore it has been paid more and more attention by people [4,5,6]. In the existing hydrogen production technology, electrolyzed water is a relatively mature technology for hydrogen production. The oxygen evolution reaction (OER) is the critical half reaction and needs more efficient electrocatalysts [7,8,9]. So, looking for the high efficiency and low cost of the electrode material is the main factor that affects the efficiency and energy consumption of electrolyzed water [10,11].

At present, the main electrode materials are precious metals and their oxides (Ru, Ir, IrO_2_, RuO_2_, etc.) [12,13,14]. However, whether precious metals or their oxides, the anode polarization is serious under strong alkaline conditions, and it is easy for the electrode to be corroded. Also, the reserves are limited and the price is high [15,16,17,18]. Therefore, it is committed to the use of non-precious metals instead of precious metals electrocatalysts for OER [19,20,21]. Transition metals such as Co, Ni, Fe, and their oxide materials, as well as Fe-Ni-, Ni-Mo-, Cu-Ni-alloy electrodes synthesized by introducing Ni(OH)_2_ onto Ni can greatly reduce overpotential of oxygen reaction and exhibit great OER performance comparable to that of noble metal catalysts [22,23,24,25,26].

However, these transition metals still face pulverization issues due to particle aggregation, low conductivity, and instability [27,28]. To overcome these obstacles, metal–organic frameworks (MOFs) with special structural properties and changes in its internal structure have been used as precursors or sacrificial templates for synthesizing single-metal oxide/sulfide nanoparticles embedded in heteroatom-doped nanoporous carbon through thermal decomposition [29,30,31,32,33]. For example, Ni-Co-based sulfide nanoparticles homogeneously embedded in N- and S-co-doped porous carbon by a one-step sulfurization and carbonization of Ni-substituted ZIF-67 simultaneously [34]. Sun et al. reported 2D Ni-Fe-MOFs (2,5-thiophene dicarboxylic acid as ligand) with high efficiency of water splitting and solar-water splitting [35]. Lin et al. reported 2D N-doped Ni-Ni_3_S_2_@carbon nanoplates (Py-1.5@SNC600) using 4,4′-bipyridine as an organic precursor for an enhanced OER electrocatalyst [36]. These reported catalysts are usually prepared by using single-ligand MOFs as precursors.

But it still faces significant challenges in achieving precise, controllable, and repeatable doping of the carbon network with heteroatoms. In this paper, we successfully obtain a multi-hole nitrogen- and sulfur-co-doped nickel-carbon material derived in situ from the dual-ligand nickel MOF through pyrolysis in an inert gas environment. The introduction of heteroatoms such as N and S in the textural properties of C can effectively modify the electrochemical behaviors and wettability of an electrode. The research results show the Ni NPs@SN-CNP exhibits superior oxygen evolution performance and a large surface area (271.9 m^2^ g^−1^) due to the synergistic effect between Ni nanoparticles and S- and N-co-doped hierarchical carbon. Furthermore, the unique morphology, nanostructure, and appropriate doping level of heteroatoms of the electrocatalyst are also important factors contributing to its superior performance. The Ni NPs@SN-CNP catalyst exhibits enhanced OER performance by combining the beneficial characteristics of NPs, SN-CNP, and the combination of NPs and SN-CNP. The higher performance of Ni NPs@SN-CNP comes from its large surface area, more catalytic sites, electron transfer, and shorten mass pathway. This work offers a new and cost-effective approach to fabricating electrocatalysts with excellent performance for the OER.

## 2. Results and Discussion

### 2.1. Structure and Morphology of the Ni NPs@SN-CNP

**Figure 1:**
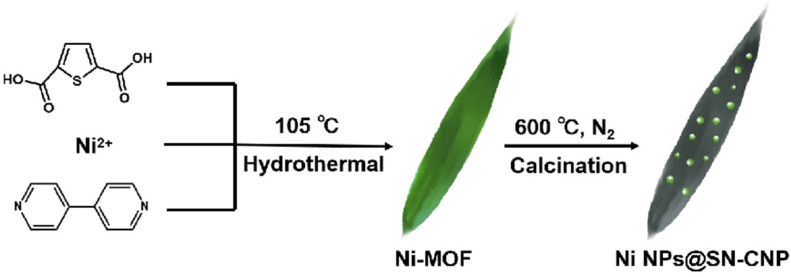
Schematic presentation of synthesis process of Ni NPs@SN-CNP.

The synthesis process of Ni NPs@SN-CNP is illustrated in Figure 1. The preparation of Ni NPs@SN-CNP involved a two-step method. Firstly, the MOF was obtained by a solvothermal method. Ni(NO_3_)_2_·6H_2_O, 4,4′-bipyridine, thiophene-2,5-dicarboxylate, and distilled water were mixed together, and the mixture was heated in an autoclave at 105 °C for 3 days. Granulated green crystals were obtained. For comparison, the Ni-MOFs were separately pyrolyzed at 500 °C, 600 °C, 700 °C, and 800 °C for 180 min each. The resulting products were represented as Ni-MOF-500, Ni NPs@SN-CNP, Ni-MOF-700, and Ni-MOF-800, respectively. The electrochemical test results reveal that the Ni NPs@SN-CNP shows the best electrochemical catalytic performance among the four resulting products. And, in the course of the pyrolysis process, the Ni (II) was reduced to metallic nickel and the ligand was carbonated under N_2_ atmosphere. XPS data will demonstrate the presence of pyridinic N and pyridine-N-oxide.

**Figure 2:**
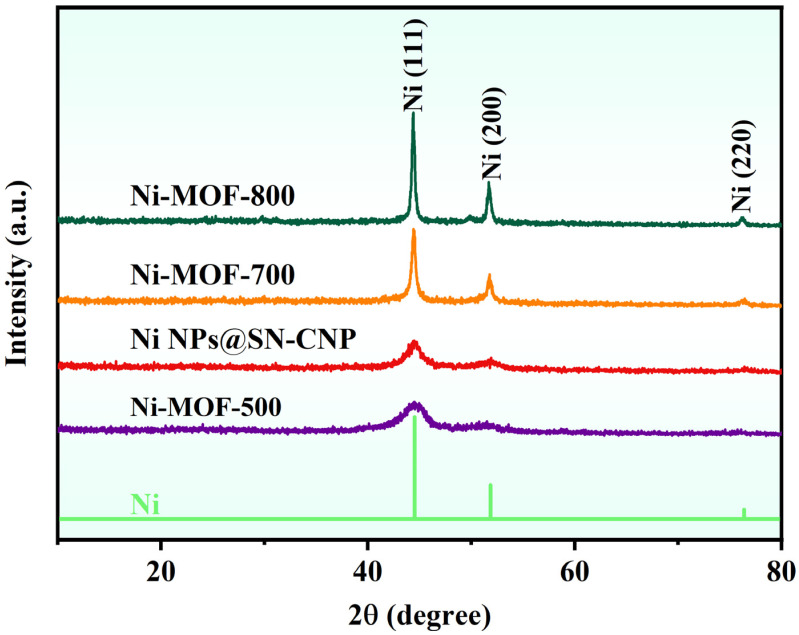
XRD pattern of Ni-MOF-500, Ni NPs@SN-CNP, Ni-MOF-700, and Ni-MOF-800.

From the XRD pattern (Figure 2), it can be seen that after pyrolysis of Ni-MOFs at 500 °C, 600 °C, 700 °C, and 800 °C, only three nickel phase diffraction peaks were exhibited (JCPDS card no. 65-2865) and located at 44.3°, 51.6°, and 75.9°, respectively. This phenomenon indicates the only crystalline species observed are related to metallic Ni in the four samples, demonstrating that the Ni-MOFs decomposes into metallic Ni at different pyrolysis temperatures.

**Figure 3:**
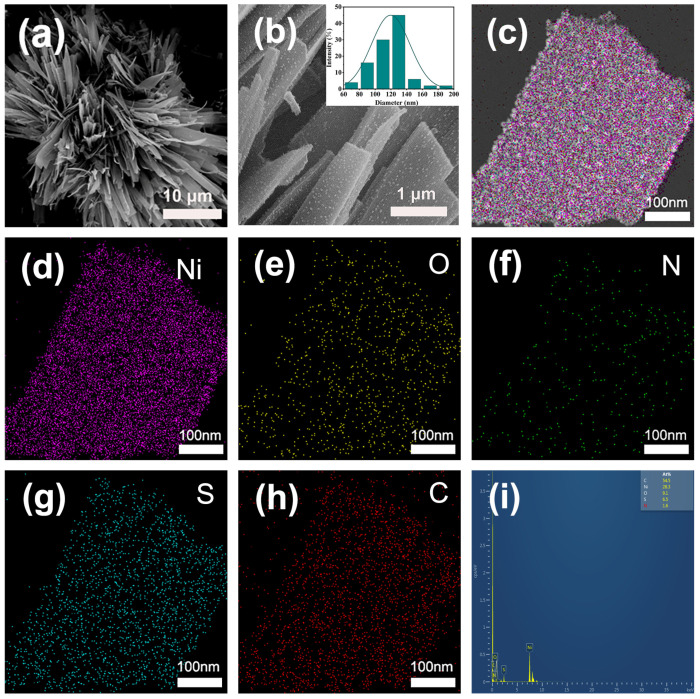
(**a**,**b**) FESEM images (inset is corresponding size distribution). (**c**) TEM and (**d**–**h**) element distribution images of Ni NPs@SN-CNP. (**i**) The spectrum of the proportion of the corresponding elements in the Ni NPs@SN-CNP.

The microstructure and morphology of the as synthesized Ni NPs@SN-CNP catalyst was characterized by a Field-Emission Scanning Electron Microscope (FESEM, Scios 2 HiVac, FEI, Hillsboro, OR, USA). Figure 3a shows that a lot of long ribbon are piling up together to form a flower-like structure. The inset of Figure 3b indicates that the average width of the carbon ribbon is 120 nm. Figure 3b shows that the surface of the ribbon is rough. Figure 3c–h shows the TEM image of Ni NPs@SN-CNP and the corresponding elemental mapping analysis, respectively. Figure 3h shows that it could be confirmed that the Ni, O, N, S, and C element are uniformly distributed, which indicates that N and S elements are doped successfully in the Ni NPs@SN-CNP. Figure 3i shows the atom percentage of the C, Ni, O, S, and N is 54.5%, 28.3%, 9.1%, 6.5%, and 1.6%.

**Figure 4:**
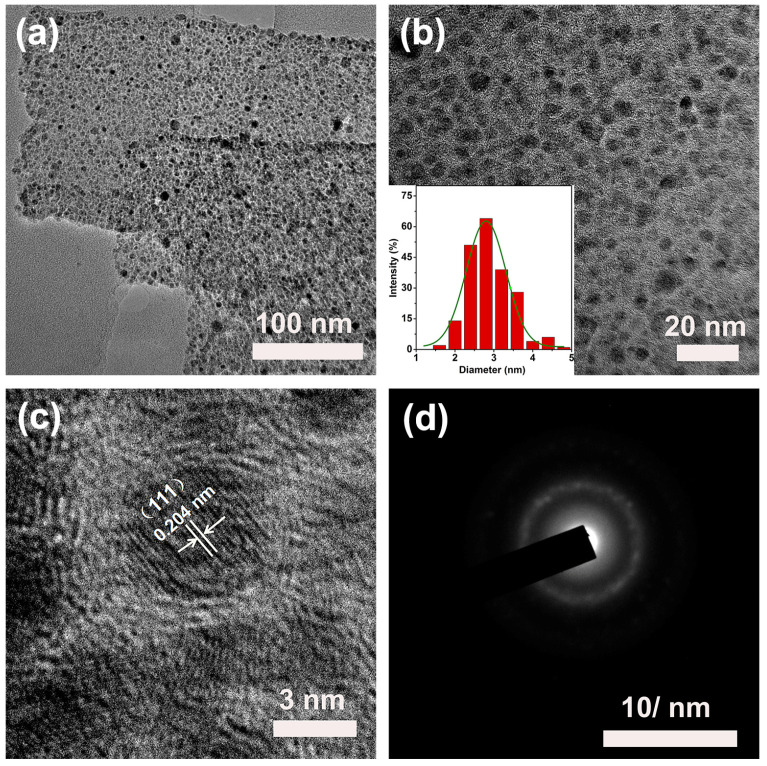
(**a**,**b**) TEM images (inset is corresponding size distribution). (**c**) High-resolution TEM (HRTEM) of the Ni NPs@SN-CNP. (**d**) SAED pattern of the Ni NPs@SN-CNP.

Figure 4a,b is the TEM images of the Ni NPs@SN-CNP catalyst. As can be seen from Figure 4a, the width of the carbon ribbon is 120 nm or so. From Figure 4b, there are many nanoparticles in the carbon ribbon. It is evident that the metallic Ni is surrounded by the carbon. The inset of Figure 4b indicates that the average diameter of the nickel nanoparticles is around 3 nm which can be further confirmed by HRTEM (Figure 4c). Additionally, the lattice spacings of 0.204 nm detected in the HRTEM image are consistent with the (111) planes of metallic Ni. The ring patterns observed from the SAED image in Figure 4 can further prove it. The results are consistent with the XRD observation. The smaller metallic nickel nanoparticles that are buried in carbon can well-prevent themselves by oxidation.

**Figure 5:**
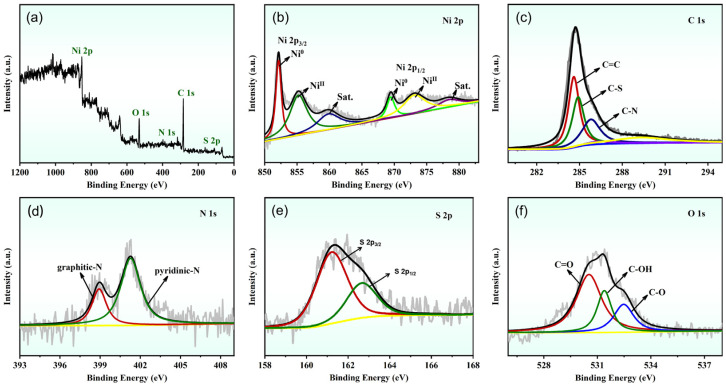
(**a**) XPS survey spectrum of high-resolution (**b**) Ni 2p, (**c**) C 1s, (**d**) N 1s, (**e**) S 2p, and (**f**) O 1s for the Ni NPs@SN-CNP.

To further ascertain the elemental composition and chemical states of the Ni NPs@SN-CNP catalyst, the XPS was used. The XPS full spectrum (Figure 5a) of Ni NPs@SN-CNP shows that there are Ni 2p, C 1s, O 1s, N 1s, and S 2p. And the relative atomic percentages of Ni, C, N, S, and O are 11.63, 69.4, 6.33, 4.9, and 7.73 in this Ni NPs@SN-CNP catalyst. Figure 5b–f shows the deconvoluted spectrums of Ni 2p, C 1s, N 1s, S 2p, and O 1s in the Ni NPs@SN-CNP catalyst. The Ni 2p spectrum (Figure 5b) consists of the main peak of Ni^0^ (852.1 eV), the other two peaks of the Ni ion are at 855.6 and 869.6 eV. The two peaks at 861.5 and 874.8 eV are satellite peaks of the Ni ion [37,38]. In composite materials, Ni NPs often reveal a partial oxidation since the samples need to be briefly handled in air to be introduced into the XPS instrument.

As shown in Figure 5c, there are three peaks in the C 1s spectrum. The peaks located at 284.9 eV and 285.8 eV. 284.6 eV correspond to C-S, C-N, and sp^2^-hybridized C=C bonds, respectively [39,40].

N 1s spectrum (Figure 5d) shows the presence of two states of N in Ni NPs@SN-CNP, including graphitic-N at 401.1 eV and pyridinic-N at 398.0 eV, both of which were reported to be active sites of OER [41,42,43,44].

From Figure 5e, the S 2p XPS spectra of the Ni NPs@SN-CNP can be fitted into two peaks. The peaks at 163.8 eV and 165.3 eV are attributed to S 2p_3/2_ and S 2p_1/2_, respectively. Their present peak area ratio is 2:1 [43]. These results confirm that carbon is co-doped with S and N, enhancing the electrocatalytic performance of the catalyst by incorporating additional active sites. The O 1s XPS spectra (Figure 5f) exhibits three peaks at 532.5 eV, 531.4 eV, and 530.7 eV, ascribed to C-O, C-OH, and C=O, respectively.

**Figure 6:**
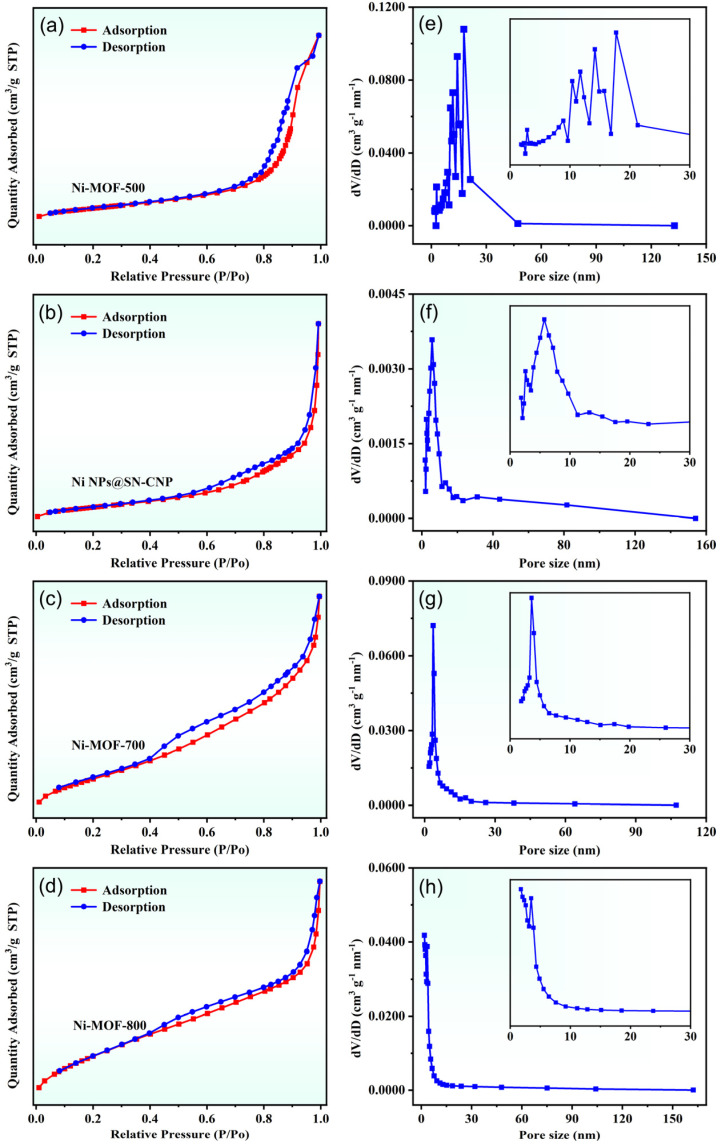
(**a**–**d**) Nitrogen adsorption–desorption isotherms and (**e**–**h**) the corresponding pore size distributions of Ni-MOF-500, Ni NPs@SN-CNP, Ni-MOF-700, and Ni-MOF-800.

The N_2_ adsorption–desorption isotherm can be used to verify the surface area and porosity of the Ni NPs@SN-CNP. As shown in Figure 6a–d, the isotherms are of typical type IV, and their surface areas are 182.8 m^2^ g^−1^ (Ni-MOF-500), 271.9 m^2^ g^−1^ (Ni NPs@SN-CNP), 169.8 m^2^ g^−1^ (Ni-MOF-700), and 169.3 m^2^ g^−1^ (Ni-MOF-800). Figure 6e–h shows that their pore sizes are centered at 10.3 nm, 11.7 nm, 14.1 nm, 17.7 nm (Ni-MOF-500), 3.0 nm, 5.7 nm (Ni NPs@SN-CNP), 3.5 nm (Ni-MOF-700), and 1.7 nm and 2.3nm (Ni-MOF-800), indicating that after calcination, the surface area of the material increases to varying degrees, and there are mesopores present in the material. The calcination at high temperature would cause the structure of MOF to collapse and the agglomerate of Ni NPs, thus seriously decreasing surface area and catalytic activity. The good catalytic performance of Ni NPs@SN-CNP can be attributed to its large surface area and mesoporous structure, which exposes abundant catalytic active sites to improve catalytic efficiency.

### 2.2. OER Performance

**Figure 7:**
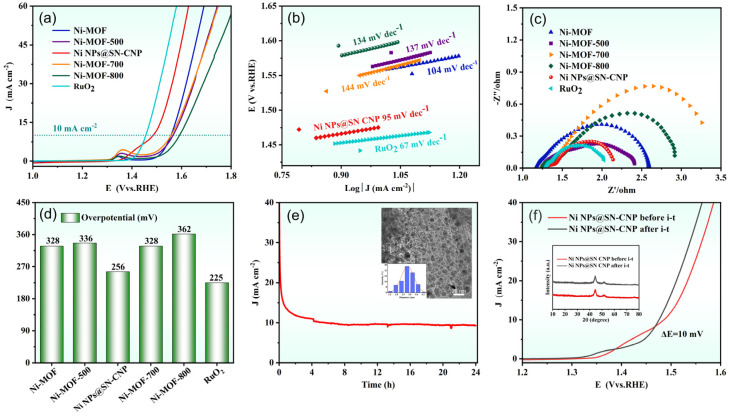
(**a**) LSV curves for the OER of Ni-MOF, Ni-MOF-500, Ni NPs@SN-CNP, Ni-MOF-700, Ni-MOF-800, and RuO_2_ catalysts in N_2_-saturated 1 M KOH. (**b**) Tafel plots of Ni-MOF, Ni-MOF-500, Ni NPs@SN-CNP, Ni-MOF-700, Ni-MOF-800, and RuO_2_ catalysts based on (**a**). (**c**) Nyquist plots of the six catalysts. (**d**) Overpotential of Ni-MOF, Ni-MOF-500, Ni NPs@SN-CNP, Ni-MOF-700, Ni-MOF-800, and RuO_2_ catalysts in N_2_-saturated 1 M KOH. (**e**) i-t curve of Ni NPs@SN-CNP at 1 M KOH and 10 mA/cm^−2^ of current density (inset: TEM image of Ni NPs@SN-CNP after i-t treated and corresponding size distribution). (**f**) LSV curves for the OER of Ni NPs@SN-CNP before and after being i-t treated (inset: XRD pattern of Ni NPs@SN-CNP before and after being i-t treated).

Using a typical three-electrode system to assess the OER performance of catalysts in N_2_-saturated 1 M KOH, as shown in Figure 7a,d, the OER LSV curves and corresponding overpotentials at 10 mA cm^−2^ (η10,OER) are for Ni-MOF (328 mV), Ni-MOF-500 (336 mV), Ni NPs@SN-CNP (256 mV), Ni-MOF-700 (328 mV), Ni-MOF-800 (362 mV), and RuO_2_ (225 mV). Ni NPs@SN-CNP has a slightly higher η10,OER than commercial RuO_2_ and lower than Ni-MOF, Ni-MOF-500, Ni-MOF-700, and Ni-MOF-800, exhibiting superior OER performance, demonstrating that 600 °C is the optimal pyrolysis condition for Ni-MOF. Figure 7b shows that the Ni NPs@SN-CNP exhibits a lower Tafel slope value (95 mV dec^−1^) than Ni-MOF (104 mV dec^−1^), Ni-MOF-500 (137 mV dec^−1^), Ni-MOF-700 (144 mV dec^−1^), and Ni-MOF-800 (134 mV dec^−1^), and is closer to commercial RuO_2_ (67 mV dec^−1^). η10,OER and Tafel slope of the Ni NPs@SN-CNP is also comparable to those reported for some non-precious metal catalysts, such as Ni10-CoS_2_ (304 mV, 98 mV dec^−1^) [45], Ni(OH)_2_@CoB (320 mV, 94 mV dec^−1^) [24], Ni-BTC (390 mV, 114 mV dec^−1^) [46], Fe-Ni·MOFNSS (258 mV, 40.8 mV dec^−1^) [47], CoNi-ZIF-67@Ti_3_C_2_T_X_ (275 mV, 65 mV dec^−1^) [48], and Ni-MOF@Fe-MOF (265 mV, 82 mV dec^−1^) [49]. These results suggest that Ni NPs@SN-CNP possesses the fast OER kinetics [50,51], which demonstrates that Ni NPs@SN-CNP has excellent OER performance. From the electrochemical impedance spectroscopy curves (Figure 7c) it can be observed that the intercept of materials is arranged in ascending order as follows: RuO_2_, Ni NPs@SN-CNP, Ni-MOF-500, Ni-MOF, Ni-MOF-800, and Ni-MOF-700. The small semi-circular diameter and closeness to RuO_2_ of Ni NPs@SN-CNP reflects the small interfacial transfer resistance during the catalytic process, facilitating electron transfer, which is in accord with the results of the faster charge transfer in the OER process and the smaller Tafel slope value [52].

Stability is an important parameter that determines the practicality of OER electrocatalysts, thus, we evaluated the stability of the Ni NPs@SN-CNP in 1 M KOH solution. As can be seen in Figure 7e, after 24 h of continuous durability testing at a current density of 10 mA cm^−2^, there was no deactivation of the catalyst. To further confirm the stability of the Ni NPs@SN-CNP, we conducted LSV analysis on a before and after stability test of Ni NPs@SN-CNP. As shown in Figure 7f, there is a negligible increase (10 mV) in overpotential after 24 h of cycling. The figures inserted in Figure 7e,f are TEM and XRD diagrams of Ni NPs@SN-CNP after stability testing, respectively, showing there is no agglomeration and material change after stability testing. The above results all indicate the excellent stability of Ni NPs@SN-CNP, providing reference for the development of stable catalysts.

**Figure 8:**
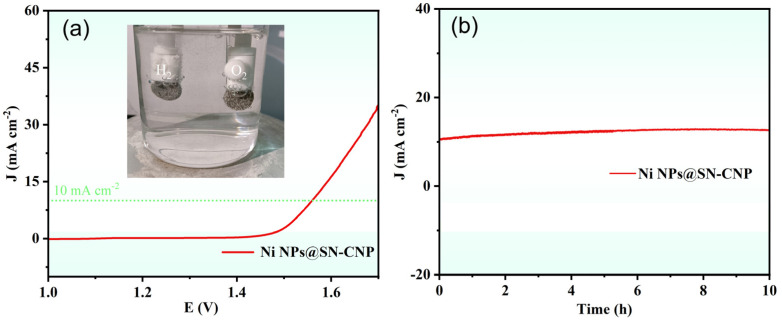
(**a**) The LSV curve of Ni NPs@SN-CNP in 1 M KOH. (Inset: the picture of H_2_ and O_2_ emerge from the Pt-C/NF electrode and Ni NPs@SN-CNP/NF electrode, respectively). (**b**) i-t curve of Ni NPs@SN-CNP at the potential of 10 mA cm^−2^ in 1 M KOH.

Based on the excellent OER performance of Ni NPs@SN-CNP, we fabricated a two-electrode system by using Pt-C/NF as the cathode and Ni NPs@SN-CNP/NF as the anode for overall water splitting in 1 M KOH. From Figure 8a, it can be clearly seen that H_2_ and O_2_ emerge from the Pt-C/NF electrode and Ni NPs@SN-CNP/NF electrode, respectively. The Ni NPs@SN-CNP/NF||Pt-C/NF cell only needs 1.56 V to achieve 10 mA cm^−2^, which is comparable to some reported catalysts, such as Ru/Ni_3_Se_4_Ni(OH)_2_/NF (1.51 V) [15], Co_0.67_Ni_0.33_P/C (1.59 V) [53], CoFeZr oxides (1.63 V) [29], and FeCoP_2_@NPPC (1.60 V) [30]. In addition, the assembled two-electrode system remained stable after 10 h of being i-t treated at the potential of 10 mA cm^−2^ (Figure 8b), indicating the good stability of Ni NPs@SN-CNP.

## 3. Materials and Methods

### 3.1. Material

Ni(NO_3_)_2_·6H_2_O, 2, 5-thiophenedicarboxylic acid, 4,4′-bipyridine, and N,N-dimethylformamide were provided by Aladdin Industrial Corporation (Shanghai, China). In this work, all chemical reagents were analytical grade and do not need further purification before use.

### 3.2. Synthesis of Ni-MOFs

In a typical synthesis procedure for Ni-MOFs, Ni(NO_3_)_2_.6H_2_O (0.2908 g), 4,4′-bipyridine (0.1562 g), and 2, 5-thiophenedicarboxylic acid (0.1722 g) were slowly added to a mixed solvent of dimethylformamide (10 mL) and distilled water (10 mL), then the turbid liquid was ultrasonicated for 30 min. Subsequently, after 3 days of continuous heating at 105 °C, filtering and washing with acetone and anhydrous ethanol several times, the bright green Ni-MOFs were synthesized.

### 3.3. Synthesis of Ni NPs@SN-CNP

A total of 0.3 g of the Ni-MOFs were placed into a porcelain boat and calcine in it under a N_2_ atmosphere at 600 °C for 3 h with a heating rate of 5 °C/min. The black Ni NPs@SN-CNP powder catalysts were obtained by natural cooling to room temperature.

As comparison, the products obtained by pyrolyzing Ni-MOFs as precursors at different temperatures (500 °C, 700 °C, 800 °C) were labeled as Ni-MOF-500, Ni-MOF-700, and Ni-MOF-800, respectively.

### 3.4. Characterizations

The surface chemical qualities of the composites were measured by X-ray photoelectron spectroscopy (XPS, 250XI, Thermofisher Scientific, Waltham, MA, USA). The structure information of the catalysts was revealed by X-ray diffractometer (XRD, D8 Advance A25, Bruker, Karlsruhe, Germany). The morphology and size data of Ni NPs@SN-CNP can be displayed by transmission electron microscope (TEM, JEM-2100, JEOL, Tokyo, Japan). The surface area and pore size distribution of the catalysts were obtained by using specific surface and pore size analysis instrument (BET, 3H-2000PS2, BeiShiDe Instrument-S&T, Beijing, China). All reagents were purchased from commercial sources and used without further treatment, unless otherwise indicated.

### 3.5. Electrochemical Measurements

The homogeneous suspension of catalyst was prepared by mixing 2 mg of catalyst or Pt-C, 1mg carbon black, 15 µL of 5wt% Nafion perfluorinated resin solution, and 485 µL of anhydrous ethanol, and the mixture was ultrasonically dispersed for approximately 30 min. Next, 25.6 µL of the suspension was uniformly dropped onto a glassy carbon RDE, which was ultrasonic cleaned with acetone and anhydrous ethanol for 10 min, respectively. The capacity of the catalyst was 0.815 mg cm^−2^. For comparison, the commercial RuO_2_-modified electrode was followed by the above-mentioned procedure. Before conducting OER testing, N_2_ is introduced into the solution for 30 min to achieve a N_2_-saturated 1 M KOH electrolyte, and N_2_ is continuously introduced during the testing progress.

Electrochemical tests were all performed on a CHI 760E electrochemical workstation, utilizing N_2_-saturated 1 M KOH as the electrolyte. A glassy carbon RDE loaded with catalyst was used as the working electrode, and the Hg/HgCl_2_ electrode (saturated KCl) and platinum wire were utilized as the reference electrode and counter electrode, respectively. Convert all measured potentials into reversible hydrogen electrode potentials (RHE) based on the Nernst equation:

E (RHE) = E (SCE) + 0.0591 × pH + 0.244

The LSV (linear sweep voltammetry) tests were conducted at a potential of −0.1 V to 1 V and a scan rate of 5 mV s^−1^. Before conducting LSV testing, all catalyst samples must undergo 40 cycles of CV testing at a potential of 0 V to 0.7 V and a scan rate of 50 mV s^−1^ for activation. The Nyquist curves were obtained at a potential of 1.56 V vs. RHE, and a frequency range from 10^−1^ to 10^5^ Hz. The stability test was conducted by chronopotentiometry (i-t) at a potential of 10 mA cm^−2^ for 24 h. The current density is calculated based on the area of the glassy carbon RDE (0.1256 cm^−2^).

Before overall water splitting test, the cathode and anode were fabricated as follows: 377 µL of homogeneous suspension of commercial Pt-C and Ni NPs@SN-CNP were uniformly dropped onto two nickel foam (NF) with a diameter of 0.6 cm, and after drying serve them as the cathode and anode for overall water splitting, respectively. The mass loading is about 2 mg cm^−2^. The LSV curve of overall water splitting was conducted at a range of 0.4 V to 2.0 V and a scan rate of 1 mV s^−1^.

## 4. Conclusions

In summary, we use a simple method for fabricating a multi-hole nitrogen- and sulfur-co-doped nickel-carbon material derived in situ from the nickel MOF through pyrolysis in an inert gas environment. The prepared Ni NPs@SN-CNP exhibits superior oxygen evolution performance with an overpotential of 256 mV at 10 mA cm^−2^ and a small Tafel slope of 95 mV dec^−1^. In addition, when operated continuously in an alkaline 1 M KOH for more than 24 h, the electrocatalyst exhibits excellent performance and stability. More importantly, the self-assembled NPs@SN-CNP/NF||Pt-C/NF water-splitting cell also displays high performance. This performance enhancement is attributed to high specific surface area, unique morphological structure, moderate doping of heteroatoms, and the nanoscale structure. In this paper, we present a simple and economical idea to fabricate carbon-based composite materials embedded with nickel doped with S and N without extra S and N sources using Ni-MOF precursors for electrocatalysis.

## Data Availability

Data are contained within the article.
